# How to avoid the inappropriate use of antibiotics in upper respiratory tract infections? A position statement from an expert panel^☆^

**DOI:** 10.1016/j.bjorl.2018.02.001

**Published:** 2018-02-25

**Authors:** Otávio Bejzman Piltcher, Eduardo Macoto Kosugi, Eulalia Sakano, Olavo Mion, José Ricardo Gurgel Testa, Fabrizio Ricci Romano, Marco Cesar Jorge Santos, Renata Cantisani Di Francesco, Edson Ibrahim Mitre, Thiago Freire Pinto Bezerra, Renato Roithmann, Francini Greco Padua, Fabiana Cardoso Pereira Valera, José Faibes Lubianca Neto, Leonardo Conrado Barbosa Sá, Shirley Shizue Nagata Pignatari, Melissa Ameloti Gomes Avelino, Juliana Alves de Souza Caixeta, Wilma Terezinha Anselmo-Lima, Edwin Tamashiro

**Affiliations:** a Universidade Federal do Rio Grande do Sul (UFRGS), Faculdade de Medicina (FAMED), Departamento de Oftalmologia e Otorrinolaringologia, Porto Alegre, RS, Brazil; b Universidade Federal de São Paulo (UNIFESP), Escola Paulista de Medicina (EPM), Departamento de Otorrinolaringologia e Cirurgia de Cabeça e Pescoço, São Paulo, SP, Brazil; c Universidade Estadual de Campinas (UNICAMP), Departamento de Otorrinolaringologia e Oftalmologia, Campinas, SP, Brazil; d Universidade de São Paulo (USP), Faculdade de Medicina (FM), Disciplina de Otorrinolaringologia, São Paulo, SP, Brazil; e Universidade de São Paulo (USP), Faculdade de Medicina (FM), Otorrinolaringologia, São Paulo, SP, Brazil; f Hospital Infantil Sabará, Otorrinolaringologia, São Paulo, SP, Brazil; g Hospital Paranaense de Otorrinolaringologia (IPO), Instituto Paranaense de Otorrinolaringologia, Curitiba, PR, Brazil; h Faculdade de Ciências Médicas da Santa Casa de São Paulo, São Paulo, SP, Brazil; i Universidade Federal de Pernambuco (UFPE), Departamento de Cirurgia, Divisão de Otorrinolaringologia, Recife, PE, Brazil; j Universidade Luterana do Brasil, Faculdade de Medicina, Porto Alegre, RS, Brazil; k Universidade de São Paulo (USP), Faculdade de Medicina (FM), São Paulo, SP, Brazil; l Hospital Albert Einstein, São Paulo, SP, Brazil; m Universidade de São Paulo (USP), Faculdade de Medicina de Ribeirão Preto (FMRP), Departamento de Oftalmologia, Otorrinolaringologia e Cirurgia de Cabeça e Pescoço, Ribeirão Preto, SP, Brazil; n Universidade Federal de Ciências da Saúde de Porto Alegre, Hospital da Criança Santo Antônio, Serviço de Otorrinolaringologia Pediátrica, Porto Alegre, RS, Brazil; o Universidade do Estado do Rio de Janeiro (UERJ), Faculdade de Ciências Médicas, Disciplina de Otorrinolaringologia, Rio de Janeiro, RJ, Brazil; p Universidade Federal de Goiás (UFG), Goiânia, GO, Brazil; q Pontifícia Universidade Católica de Goiás (PUC-GO), Goiânia, GO, Brazil; r Centro Universitário de Anápolis, Anápolis, GO, Brazil

**Keywords:** Upper respiratory tract infections, Antibiotic, Microbial drug resistance, Acute rhinosinusitis, Acute otitis media, Infecções do trato respiratório superior, Antibióticos, Resistência bacteriana a drogas, Rinossinusite aguda, Otite média aguda

## Abstract

**Introduction:**

Bacterial resistance burden has increased in the past years, mainly due to inappropriate antibiotic use. Recently it has become an urgent public health concern due to its impact on the prolongation of hospitalization, an increase of total cost of treatment and mortality associated with infectious disease. Almost half of the antimicrobial prescriptions in outpatient care visits are prescribed for acute upper respiratory infections, especially rhinosinusitis, otitis media, and pharyngotonsillitis. In this context, otorhinolaryngologists play an important role in orienting patients and non-specialists in the utilization of antibiotics rationally and properly in these infections.

**Objectives:**

To review the most recent recommendations and guidelines for the use of antibiotics in acute otitis media, acute rhinosinusitis, and pharyngotonsillitis, adapted to our national reality.

**Methods:**

A literature review on PubMed database including the medical management in acute otitis media, acute rhinosinusitis, and pharyngotonsillitis, followed by a discussion with a panel of specialists.

**Results:**

Antibiotics must be judiciously prescribed in uncomplicated acute upper respiratory tract infections. The severity of clinical presentation and the potential risks for evolution to suppurative and non-suppurative complications must be taken into ‘consideration’.

**Conclusions:**

Periodic revisions on guidelines and recommendations for treatment of the main acute infections are necessary to orient rationale and appropriate use of antibiotics. Continuous medical education and changes in physicians’ and patients’ behavior are required to modify the paradigm that all upper respiratory infection needs antibiotic therapy, minimizing the consequences of its inadequate and inappropriate use.

## Resumo

**Introdução:**

A resistência bacteriana a antibióticos nos processos infecciosos é um fato crescente nos últimos anos, especialmente devido ao seu uso inapropriado. Ao longo dos anos vem se tornando um grave problema de saúde pública devido ao prolongamento do tempo de internação, elevação dos custos de tratamento e aumento da mortalidade relacionada às doenças infecciosas. Quase a metade das prescrições de antibióticos em unidades de pronto atendimento é destinada ao tratamento de alguma infecção de vias aéreas superiores, especialmente rinossinusites, otite média aguda supurada e faringotonsilites agudas, sendo que uma significativa parcela dessas prescrições é inapropriada. Nesse contexto, os otorrinolaringologistas têm um papel fundamental na orientação de pacientes e colegas não especialistas, para o uso adequado e racional de antibióticos frente a essas situações clínicas.

**Objetivos:**

Realizar uma revisão das atuais recomendações de utilização de antibióticos nas otites médias, rinossinusites e faringotonsilites agudas adaptadas à realidade nacional.

**Método:**

Revisão na base PubMed das principais recomendações internacionais de tratamentos das infecções de vias aéreas superiores, seguido de discussão com um painel de especialistas.

**Resultados:**

Os antibióticos devem ser utilizados de maneira criteriosa nas infecções agudas de vias aéreas superiores não complicadas, a depender da gravidade da apresentação clínica e dos potenciais riscos associados de complicações supurativas e não supurativas.

**Conclusões:**

Constantes revisões a respeito do tratamento das principais infecções agudas são necessárias para que sejam tomadas medidas coletivas no uso racional e apropriado de antibióticos. Somente com orientação e transformações no comportamento de médicos e pacientes é que haverá mudanças do paradigma de que toda infecção de vias aéreas superiores deva ser tratada antibióticos, minimizando por consequência os efeitos de seu uso inadequado.

## Introduction

Bacterial resistance to antibiotics in infectious processes has been increasing in recent years and has become a serious public health problem.^1^

In October 2017, the World Health Organization (WHO) stated that bacterial resistance to antibiotics is one of the main health problems worldwide, as it prolongs hospital length of stay, increases treatment costs and, even more seriously, considerably increases mortality related to infectious diseases.^2^ According to the WHO, inappropriate antibiotic use is considered the main reason for the generation of bacterial resistance to antibiotics. In developed countries such as the USA and Canada, it is estimated that 30–50% of antibiotic prescriptions are inappropriate.^3^, ^4^, ^5^ Furthermore, approximately 50% of all antibiotic prescriptions aim at the treatment of upper respiratory infections, especially rhinosinusitis, suppurative acute otitis media, and acute pharyngotonsillitis.^4^ In this context, the role of the otorhinolaryngologist is crucial when advising patients and non-specialists regarding the adequate and rational use of antibiotics, particularly for these clinical conditions.^3^, ^6^, ^7^ A broad review of indications and forms of antibiotic use is required for the very diverse infectious conditions, with evidence-based collective actions. Hence, several countries have adopted public policies to considerably reduce inadequate antibiotic prescriptions. In one such example, the National Action Plan to fight antibiotic-resistant bacteria, was launched in the United States in 2015, aiming to reduce by 50% inadequate antibiotic prescription by 2020.^4^

The present document reflects the concern of the Brazilian Association of Otorhinolaryngology and Cervical-Facial Surgery (ABORL-CCF) to adequately guide physicians on the appropriate antibiotic prescription in cases of acute otitis media, acute rhinosinusitis and acute pharyngotonsillitis, aiming to change the behavior of physicians and patients in order to break the paradigm that all upper respiratory infections should be treated with antibiotics, thus minimizing the effects of their inappropriate use.

## Acute Otitis Media

Acute otitis media (AOM) is a common disease in early childhood (with a peak incidence between 6 months and 2 years), but it also affects older children and, less commonly, adolescents and adults.

To attain the diagnosis, it should be considered that hyperemia, diminished tympanic membrane translucency or the presence of retropharyngeal fluid alone, without bulging or otorrhea, are not signs that differentiate AOM. Tympanic membrane bulging is the most reliable finding.^8^ AOM caused by pneumococcus is more associated with important tympanic membrane alterations (mainly bulging), fever and otalgia, whereas that caused by *H. influenzae* is more associated with ocular symptoms (purulent conjunctivitis).^9^

Pathogenic bacteria are isolated in approximately 70% of cases of AOM, including *Haemophilus influenzae*, *Streptococcus pneumoniae*, and *Moraxella catarrhalis*. The pneumococcus, previously the most prevalent agent, has been supplanted by *H. influenzae* in most countries that have implemented mass vaccination of the population, such as in the USA.^10^ Although we do not have national data on middle ear secretion culture in cases of AOM, a recent Brazilian study has demonstrated an increase in the isolation of non-typeable *H. influenzae* and a reduction of *S. pneumoniae* in the rhinopharynx of children that received the 10-valent pneumococcal conjugate vaccine, suggesting that the same process that occurred in the USA after the introduction of mass vaccination may also be occurring in Brazil.^11^

Approximately 30% of middle ear cultures yield no bacterial isolates or show the presence of viruses, such as Influenza, parainfluenza, rhinovirus and respiratory syncytial virus, reinforcing the important role of viruses in the etiology of AOMs.

In the USA, antibiotic prescription for treatment of childhood AOM is more frequent than for any other infectious disease. It is estimated that more than 80% of the diagnosed cases are immediately treated with antibiotics in most countries, but for rare exceptions, as in the Netherlands (31.2% of cases).^12^, ^13^

The natural history of non-severe AOMs shows that the cure of this condition usually occurs regardless of the use of antibiotics. Fortunately, non-severe cases represent the vast majority of patients that seek pediatric medical offices or emergency care units. Although there is indeed an additional benefit in prescribing antibiotics to resolve the AOM picture, this benefit is a modest one, increasing the resolution rate by only 12–14% when compared to placebo (92–94% with ATB use vs. 80% without ATB use). Therefore, 7–9 children with AOM need to be treated to obtain the additional benefit of antibiotic use in one of them.^14^

A recent meta-analysis that evaluated pain reduction with antibiotic use in AOM through an analog scale did not show a significant reduction when compared to placebo in the first 24 h, with a beneficial effect (although not clinically significant) appearing only after the 2nd day of treatment. For the specific assessment of pain reduction, the most favorable Number Needed to Treat (NNT) occurs at the end of 10–12 days of treatment, when it reaches 7 (NNT = 20 between the 2nd and 3rd days; NNT = 16 between the 4th and 7th days).^15^

Antibiotics have a beneficial effect in reducing effusion up to 6 weeks after the end of treatment, preventing early recurrence of AOM and perforation onset, but they are all are clinically modest. However, antibiotic use does not prevent more serious complications, or the presence of effusion or late recurrences (after 3 months). On the other hand, adverse effects such as vomiting, diarrhea and skin rash are significantly more frequent in children who receive antibiotics.^15^

In two studies that performed culture by tympanic membrane puncture before and after the treatment, a more accurate way to diagnose and evaluate bacteriological cure,^16^ 3–7% of patients who had negative culture between day 3 and day 7 failed to respond to the antibiotic. On the other hand, of those who maintained a positive culture, the failure rate was 37–38%, which means that 62–63% of the patients achieved clinical cure, even while maintaining a positive culture between day 3 and day 7.^16^, ^17^

Much of the criticism aimed at the evidence of the small effect of antibiotics is based on the possibility of diagnostic error when including children in the clinical trials without the disease or without bacterial AOM. The definitive diagnosis of AOM is based on otoscopy, which in young children can be extremely difficult.^12^

### Treatment

The use of analgesics and antipyretics should be immediate, since antibiotics take up to 48 h to relieve the picture of fever and otalgia. Among the most commonly used analgesics are dipyrone, acetaminophen (paracetamol) and ibuprofen.

Because of the greater benefit of using antibiotics on some occasions, the American Academy of Pediatrics recommends the use of antibiotics in the following situations^18^, ^19^:

• Children younger than 6 months.
• Children older than 6 months with severe disease (moderate to severe otalgia for more than 48 h, or temperature ≥ 39 °C).
• Bilateral AOM (NNT = 5).
• Presence of otorrhea (NNT = 3).

As for the cases that require treatment with antibiotics, these should cover the most commonly involved bacteria. The recommended treatment for uncomplicated cases is amoxicillin (45 mg/kg/day, divided into two or three doses), which may be associated with beta-lactamase inhibitors in patients with aggravating comorbidities or suspected/confirmed resistant infections (for instance, culture proving resistance, previous poor outcome with the drug, recent antibiotic use) (Table 1).^18^, ^20^, ^21^

**Table 1:** Antibiotics recommended for the treatment of acute otitis media.^22^

Initial antibiotic treatment at the time of diagnosis or after observation		Antibiotic treatment after 48–72 h of initial treatment failure	
First-line treatment	Alternative treatment	First-line treatment	Alternative treatment
Amoxicillin (45–90 mg/kg/day)	Cefuroxime (30 mg/kg/day) (In allergic reaction to non-type I penicillin)	Amoxicillin–clavulanate (45–90 mg/kg/day of amoxicillin with 6.4 mg/kg/day of clavulanate)	Ceftriaxone 3 days, or Clindamycin (30–40 mg/kg/day) with or without second- or third-generation cephalosporin Vancomycin IV
Or		Or	
Amoxicillin–clavulanate^a^ (45–90 mg/kg/day of amoxicillin with 6.4 mg/kg/day of clavulanate)	Clarithromycin (15 mg/kg weight/day) (In allergic reaction to type I penicillin)	Ceftriaxone (50 mg/kg/day IM or IV for 3 days)	Clindamycin + second- or third-generation cephalosporin
	Ceftriaxone (50 mg/kg/day IM or IV for 1–3 days)		Consult specialist
			Tympanocentesis^b^

a It can be considered in patients that received amoxicillin in the previous 30 days or have otitis-conjunctivitis syndrome.

b Find an otorhinolaryngologist for tympanocentesis/drainage/secretion collection for culture and antibiogram.

The American Academy of Pediatrics recommends the use of amoxicillin at doses of 90 mg/kg/day, associated or not with potassium clavulanate.^18^ However, the intermediate resistance of pneumococcus in our country is still low and thus, this measure is not justified as the first option in Brazil.^22^

Regarding treatment duration, the indication of using the antibiotic for at least 10 days remains, especially for more severely affected patients with the risk characteristics that indicate the need for antibiotic treatment (for instance, bilateral disease and otorrhea).^23^ Initial studies even showed some optimism regarding shorter treatments (5–7 days), with the potential advantages of producing fewer gastrointestinal side effects and decreasing the emergence of resistant strains. However, recent studies have shown the superiority of the 10-day treatments over shorter ones, with the same incidence of adverse effects.^23^

For patients with non-severe allergy to penicillins, second- or third-generation cephalosporins, clindamycin and macrolides, especially clarithromycin, may be used. Azithromycin and cefaclor should not be used, due to the high resistance index. Sulfa drugs should be avoided due to the low therapeutic efficacy in children.^24^

For adults, the antibiotic therapy recommendations are similar to the options used in acute bacterial rhinosinusitis (Table 2).

**Table 2:** Treatment indicated by the Expert Opinion of the Brazilian Academy of Rhinology for the treatment of uncomplicated bacterial ARS and extrapolated to the treatment of bacterial otitis media in adults.

Main antibiotic options	Dose and posology	Time of treatment^a^	Considerations
Amoxicillin	500 mg, 3×/day	7–14 days	Preferred antibiotic agent in patients with no suspected or confirmed bacterial resistance, with no prior use of antibiotics in the last 30 days for the same clinical picture.
Amoxicillin	875 mg, 2×/day	7–14 days	Preferred antibiotic agent in patients with no suspected or confirmed bacterial resistance, with no prior use of antibiotics in the last 30 days for the same clinical picture.
Amoxicillin-Clavulanate	500 mg/125 mg, 3×/day	7–14 days	Indicated for β-lactamase producing bacteria. Diarrhea occurs in 1–10% cases
Amoxicillin-Clavulanate	875 mg/125 mg, 2×/day	7–14 days	Indicated for β-lactamase producing bacteria. Diarrhea occurs in 1–10% cases
Cefuroxime Axetil	250–500 mg, 2×/day	7–14 days	Spectrum of action similar to that of amoxicillin-clavulanate. An option in cases of non-anaphylactic allergic reactions to penicillins. Evidence of increased induction of bacterial resistance in relation to penicillins.^25^

Option in those allergic to β-lactams	Dose and posology	Time of treatment^a^	Considerations
Clarithromycin	500 mg, 2×/day	7–14 days	Consider high resistance. Contraindication for the concomitant use of statins
Levofloxacin	500 mg, 1×/day	5–7 days	The Food and Drug Administration (FDA) determines that the prescription of fluoroquinolones to patients with bacterial ARS should occur only when there are no other antibiotic treatment options, as the risks outweigh the benefits in these cases.^b^
Levofloxacin	750 mg, 1×/day	5–7 days	
Moxifloxacin	400 mg, 1×/day	5–7 days	
Doxycycline	100 mg, 2×/day	7–14 days	Photosensitivity reaction

Options in therapeutic failure cases^c^	Dose and posology	Time of treatment	Considerations
Amoxicillin	1000 mg, 3×/day	7–14 days	Exception conduct proposed by some specialists, based on microbiological knowledge, without proven clinical evidence. Consider exacerbated gastrointestinal effects.
High-dose Amoxicillin + Clavulanate	2000 mg Amx/125 mg Clav, 2×/day	7–14 days	Exception conduct proposed by some specialists, based on microbiological knowledge, without proven clinical evidence. Consider exacerbated gastrointestinal effects.
Levofloxacin	750 mg, 1×/day	5–7 days	The Food and Drug Administration (FDA) determines that the prescription of fluoroquinolones to patients with bacterial ARS should occur only when there are no other antibiotic treatment options, as the risks outweigh the benefits in these cases.^b^
Moxifloxacin	400 mg, 1×/day	5–7 days	
Clindamycin	300 mg, 3–4×/day	7–10 days	Option in case of suspected infection by anaerobic bacteria or *S. aureus*. Take with 300 mL of water due to risk of esophageal lesion. Precaution: risk of membranous pseudocolitis and diarrhea caused by *Clostridium difficile*.

a There is a tendency to use antibiotic therapy for less time with equal effectiveness aiming to minimize side effects and bacterial resistance generation.

b It should be considered individually, according to disease severity.

c Absence of response or clinical worsening after 48–72 h of treatment.

**Figure 1:**
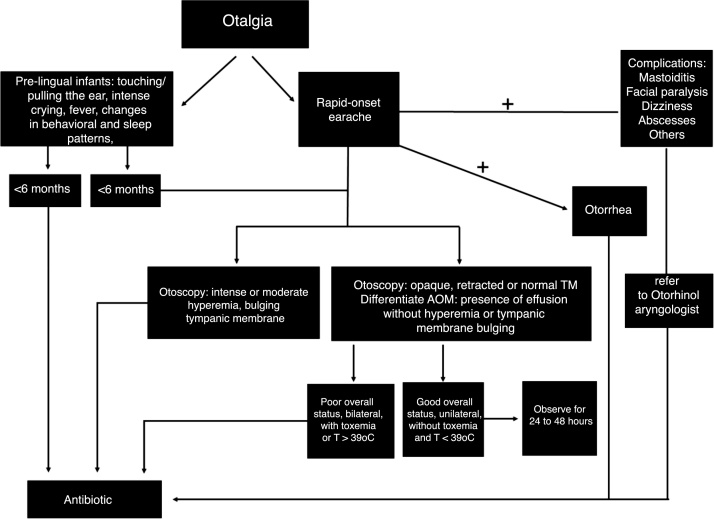
Flowchart of the diagnosis and treatment of acute otitis media.

Complications of AOMs may include tympanic membrane rupture, mastoiditis, meningitis, subperiosteal abscesses, intracranial abscesses, subdural abscesses, dural sinus thrombosis, labyrinthitis, petrositis, facial paralysis and sepsis. It is noteworthy that the early-onset of antibiotic therapy does not prevent the occurrence of suppurative complications, since most individuals with complications were receiving some type of antibiotic. In the presence of complications, tympanocentesis (with or without the insertion of a ventilation tube) should be performed whenever possible, aiming to aspirate secretions and collect material for culture. Fig. 1 summarizes the treatment flowchart for patients with AOM.

## Acute Rhinosinusitis

Acute rhinosinusitis (ARS) is inflammation of the nasal mucosa and paranasal sinuses that usually presents with two or more of the following symptoms, such as rhinorrhea and/or nasal obstruction (with one of them being mandatory), facial pain and olfactory alterations. In pediatric patients, coughing is a rather common symptom, more common than olfactory alterations (Table 3).^26^

**Table 3:** Cardinal symptoms of rhinosinusitis.

Main symptoms	Associated symptoms
Rhinorrhea	Facial pain
Nasal obstruction	Changes in olfaction Coughing (children)

The American guidelines for rhinosinusitis (2015) point out that purulent rhinorrhea is the most reliable symptom for its diagnosis. Thus, the presence of two or more symptoms is considered for the diagnosis of ARS, with the presence of purulent rhinorrhea being mandatory, associated with nasal congestion and/or facial pain.^27^

### Viral or Bacterial?

ARS cases usually start as a viral infection. Most of these cases show spontaneous resolution within 7–10 days, with clear improvement after 5 days of evolution. Only 0.5–2% of the cases show evolution to bacterial ARS in adults, and 5–13% of cases in children.^28^

**Figure 2:**
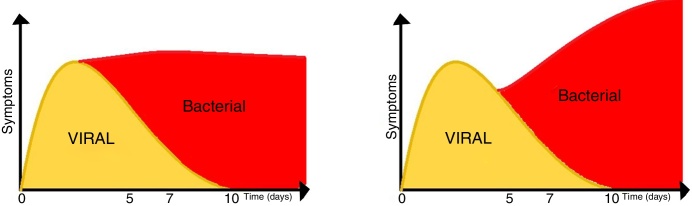
Evolution of acute bacterial rhinosinusitis after a viral illness.

Adapted from the American Guidelines for Rhinosinusitis, 2015.

According to the American guidelines for rhinosinusitis (2015), we should consider the diagnosis of acute bacterial rhinosinusitis (ABRS) when the cardinal symptoms persist for more than 10 days, with no evidence of improvement in the short term, or even symptom worsening after a period of initial improvement, which is called double-worsening (Fig. 2).^27^

**Figure 3:**
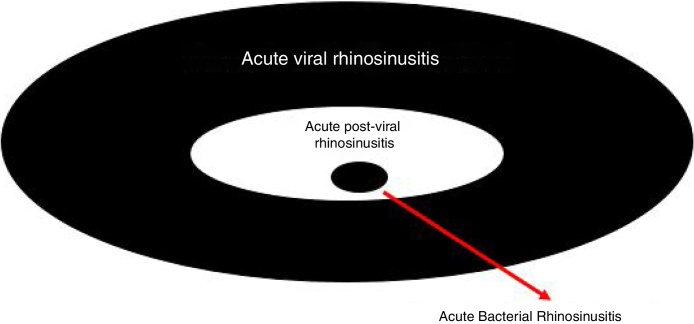
Representativeness of acute viral rhinosinusitis developing into acute post-viral rhinosinusitis or, eventually, acute bacterial rhinosinusitis, according to EPOS (2012).

**Figure 4:**
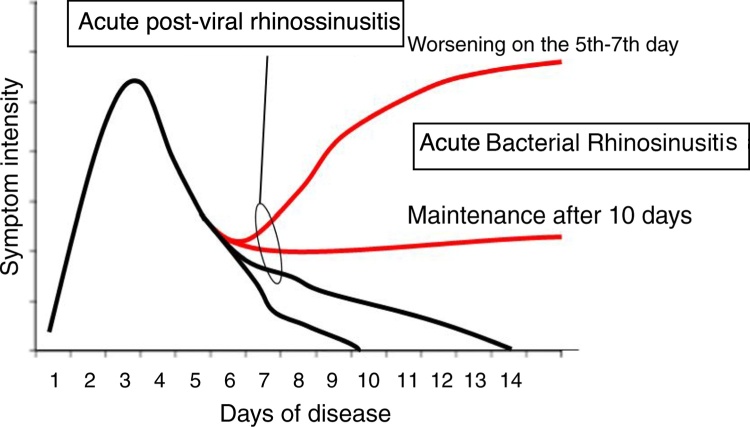
Evolution of acute rhinosinusitis.

Modified from EPOS 2012.

According to the Brazilian (2015) and European Guidelines for Rhinosinusitis (EPOS 2012 – European Position Paper on Rhinosinusitis and Nasal Polyps), cases that do not clearly show as viral (duration up to 10 days, with evident improvement after the 5th day) are called post-viral. Among post-viral pictures, the diagnosis of ABRS should be considered in patients with symptoms for more than 10 days and who have at least 3 of the following criteria (Figure 3, Figure 4)^26^, ^29^:

• Worsening after a milder initial phase.
• Predominantly unilateral rhinorrhea and/or purulent posterior rhinorrhea.
• Severe facial pain, mainly unilateral.
• Fever ≥ 38.3 °C.
• Increased values of inflammatory markers (ESR, CRP) (in practice, blood tests are rarely required for the differential diagnosis of acute rhinosinusites).

### Warning signs

The complications of ARS are extremely rare. They occur when the infection extends beyond the borders of the paranasal sinuses. It is estimated that 1 complication occurs in every 12,000 episodes of ARS in children, and 1 complication for every 32,000 episodes in adults, mandatorily requiring the use of antibiotics in these situations.^30^

Some warning signs should be observed, which indicate the possible presence of ARS complications (Table 4).

**Table 4:** Warning signs for ARS complications.

Orbital changes
Visual changes
Intense frontal pain
Frontal bulging
Signs of meningitis
Focal neurological signs
Decrease in the level of consciousness

### Treatment

Symptomatic treatment is extremely important for quality of life improvement and can be instituted in all ARS cases. The medication should be chosen according to the patient's most intense symptoms. Hence, the physician should personalize the treatment for each patient, avoiding the use of “standard prescriptions”.

Analgesics, non-steroidal anti-inflammatory drugs (NSAIDs) and topical or systemic decongestants are good therapeutic options, as they treat symptoms that bring great discomfort, such as pain, malaise, and nasal obstruction. The symptomatic relief produced by these medications may reduce the need for antibiotics in such situations. However, such medications should be used sparingly, since NSAIDs and topical and systemic decongestants are associated with a high rate of significant side effects.^27^

Nasal lavage with saline solution or hypertonic solution is recommended for the treatment of ARS, whether viral or bacterial, as it contributes to the reduction of symptoms and faster disease resolution. Currently, there are conflicting data on the use of the hypertonic solution, in addition to being more uncomfortable for the patient, as it can cause a burning sensation and lower treatment adherence. Thus, nasal lavage with isotonic solutions has been more often recommended for this purpose.^30^, ^31^

## Acute Viral Rhinosinusitis

### *Pelargonium sidoides*

Antiviral medications can be used in acute viral rhinosinusitis, such as *Pelargonium sidoides.* This phytomedicine increases the immune response to infection by decreasing viral replication. Ideally, it should be used within the first 48 h of the viral picture onset. Patients taking anticoagulants should avoid using it.^29^

### Topical Corticosteroids (CS)

Topical CS have a high local anti-inflammatory effect, with a low rate of side effects. They aid in reducing nasal mucosa edema, improving obstructive symptoms and contributing to the patency of the sinus drainage ostia. They also reduce mucus production and neurogenic inflammation, decreasing symptoms such as sneezing and nasal pruritus.

The European guidelines (EPOS 2012) recommend the use of topical CS in post-viral and bacterial acute rhinosinusitis with a double dose, aiming at the above mentioned beneficial effects.^29^ The American guidelines (2015), however, consider the use of topical CS even in acute viral rhinosinusitis.^29^

## Acute Bacterial Rhinosinusitis

### Topical Corticosteroids

In cases of acute bacterial rhinosinusitis (ABRS), the use of topical CS is recommended due to the abovementioned anti-inflammatory effects. Moreover, there is evidence that the use of topical CS has similar efficacy to the use of antibiotic alone in milder ABRS cases, which could spare the use of antibiotics. This practice should be encouraged to avoid the abusive use of antibiotics in milder situations. Similar to post-viral rhinosinusitis, double doses of topical corticosteroids tend to produce more significant beneficial effects, although the benefit of treatment is still a modest one.^27^, ^29^

In patients with severe symptoms, especially pain, oral corticosteroids may be prescribed for a short period.^29^ It is important to note that intramuscular long-lasting corticosteroid injections are not recommended.

### Antibiotics

For patients who meet the criteria for ABRS (approximately 0.5–2% of total AVRS), the use of antibiotics may be recommended. According to EPOS, mild cases can be initially treated only with measures recommended for post-viral ARS and reevaluated within 48–72 h, whereas in more severe cases they should receive antibiotic therapy.^29^

According to the American guidelines, the doctor may choose to immediately prescribe an antibiotic for ABRS or perform an initial treatment of ABRS with delayed prescription of antibiotics (watchful waiting). In this case, patients start the treatment with topical CS and nasal lavage with saline solution, but receive the prescription for the antibiotic, although they would be advised to wait to start its use (“wait-and-see” prescription). The antibiotic should be started if there is no improvement in 7 days or if there is worsening of the condition at any time. It is important to emphasize that this approach should be used only in cases that meet the criteria for ABRS and should not be used in viral cases or even those with diagnostic doubt.

The bacterial agents most often implicated in ABRS are: *S. pneumoniae*, *H influenzae* and *M. catarrhalis*. National data on antibiotic sensitivity are shown in Table 5.

**Table 5:** National profile of antibiotic sensitivity according to some isolated agents.

Microbiota	Drug	Sensitivity
*Streptococcus* spp. (except in meningitis)	Penicillin	93% (>5 years)
	Sulfamethoxazole/Trimethoprim	66%
*Haemophilus influenzae*	Ampicillin	86.5%
	Sulfamethoxazole/Trimethoprim	75%

*Source*: SIREVA 2014.

**Figure 5:**
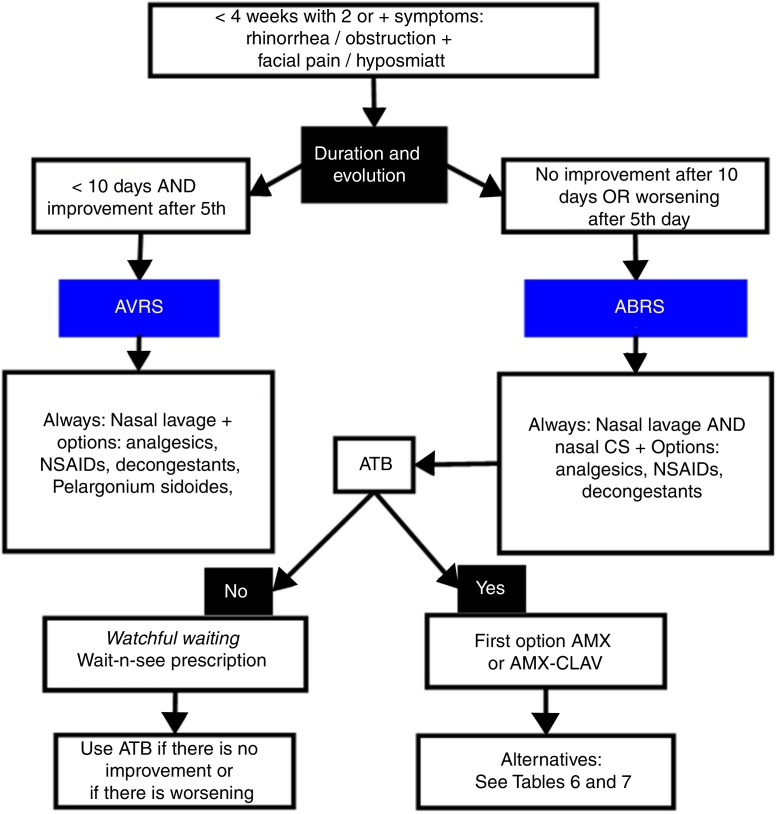
Evaluation flow chart according to the presence of signs and symptoms, aimed to elucidate the probable etiological diagnosis and its treatment.

Based on the national sensitivity profile of these microorganisms, the recommendation for the choice of antimicrobial medication is shown in Table 2, Table 6. Fig. 5 shows a flowchart for the rational evaluation of antibiotic use in the most diverse presentations of ARS.

**Table 6:** Antibiotics recommended in the treatment of ABRS in the pediatric population.

Initial antibiotic treatment at the time of diagnosis or after observation		Antibiotic treatment after 48–72 h of initial treatment failure	
First-line treatment	Alternative treatment	First-line treatment	Alternative treatment
Amoxicillin (45–90 mg/kg/day)	Cefuroxime (30 mg/kg/day) (In allergic reaction to non-type I penicillin)	Amoxicillin–clavulanate (45–90 mg/kg/day of amoxicillin with 6.4 mg/kg/day of clavulanate)	Ceftriaxone 3 days, or Clindamycin (30–40 mg/kg/day) with or without second- or third-generation cephalosporin Vancomycin IV
Or		Or	
Amoxicillin–clavulanate^a^ (45–90 mg/kg/day of amoxicillin with 6.4 mg/kg/day of clavulanate)	Clarithromycin (15 mg/kg weight/day) Sulfamethoxazole/Trimethoprim (In allergic reaction to type I penicillin)	Ceftriaxone (50 mg/kg/day IM or IV for 3 days)	Clindamycin + second- or third-generation Cephalosporin
	Ceftriaxona (50 mg/kg/day IM or IV for 1–3 days)		Consult specialist

a It can be considered as an option in children who received amoxicillin in the last 30 days or in areas with high bacterial resistance to amoxicillin.

The Brazilian guidelines and EPOS 2012 classify this situation as post-viral ARS, and post-viral conditions that meet 3 of the following criteria are considered bacterial:

• Worsening after a milder initial phase.
• Predominantly unilateral rhinorrhea and/or frankly purulent post-nasal drip.
• Severe local pain, mainly unilateral.
• Fever > 38 °C.
• Increased inflammatory markers (ESR, CRP).

## Acute Pharyngotonsillitis

Acute pharyngotonsillitis is a disease characterized by signs and symptoms related to the inflammation of the pharynx and Waldeyer's lymphatic ring structures and may be associated with a wide range of regional and/or systemic symptoms.

Most cases of pharyngotonsillitis is caused by viruses (70% of cases), usually with diffuse and mild odynophagia, low or no fever, coughing, hoarseness, nasal congestion, rhinorrhea, presence of ulcerated or vesicular lesions on the oral mucosa, insidious evolution, among others.^32^, ^33^

As for the bacterial cases, which correspond to a lower percentage in all age groups (less than 30% of the cases), usually show more severe odynophagia, high fever (>38.5 °C), lymph nodes larger than 1 cm, tonsillar and pharyngeal edema and exudate, palatal petechia, scarlatiniform rash, isolated abdominal pain and abrupt symptom onset. Among the main bacterial agents involved in acute pharyngotonsillitis, *S. pyogenes* (Lancefield group A beta-hemolytic streptococcus) stands out because of its high prevalence in the age range of 3–15 years (up to 2/3 cases), more invasive forms with systemic effects and, especially, its risk of developing rheumatic fever.

Due to its relevance for the treatment, the differentiation of acute pharyngotonsillitis caused by *S. pyogenes* plays an important role. For etiological determination, microbial identification through oropharynx culture is still considered the gold standard test (with sensitivity between 60–90% and specificity between 65–95%). Considering the non-negligible costs for its performance, as well as the time required to obtain results (48–72 h), such factors limit its routine use in cases of acute pharyngotonsillitis. An alternative test is the rapid strep test (RST) to detect streptococcal antigens, which has sensitivity and specificity similar to those of culture tests.^33^, ^34^ Although it has a great advantage regarding the time to obtain the results (approximately 15 min), this test still has a relatively high cost, which makes it impossible to apply this routine test at public services. Additionally, positive results in these tests do not allow the differentiation of the etiologic cause in chronic carriers of *S. pyogenes*.

In daily practice, other validated and reproducible clinical methods in different age groups have been used to identify the probability of *S. pyogenes* infection. One is the modified Centor clinical score (McIsaac), which includes history and physical examination characteristics (Table 7).

**Table 7:** Probability of pharyngotonsillitis by *S. pyogenes* according to the modified Centor criteria (McIsaac).^33^

Modified Centor criteria (McIsaac)	
Variable	Score
Fever > 38 °C	+1
No coughing	+1
Anterior cervical adenopathy > 1 cm	+1
Tonsillar exudate or edema	+1
Age 3–14 years	+1
Age 15–44 years	0
Age ≥ 45 years	−1

Total score	Probability of *S. pyogenes*
≤0 points	∼2.5%
1 points	∼6–7%
2 points	∼15%
3 points	∼30–35%
≥4 points	∼50–60%

Although the modified Centor score is one of the most frequently used worldwide, it still lacks good sensitivity for detection, with moderate positive predictive value. Despite these limitations, the two scoring ends (≤1 or ≥4 points) have been used as discriminators of low probability and high probability of infection by *S. pyogenes*, respectively. Therefore, for patients with a low probability of streptococcal infection (total score ≤ 1 in the modified Centor score), as well as in the more likely clinical settings (score ≥ 4 in the modified Centor score), there would be no need to routinely use the test.^34^ Patients with a moderate probability of streptococcal infection (total score of 2–3 in the modified Centor score) should ideally be submitted to culture or rapid antigen detection test for treatment decision-making.

It is noteworthy that the Modified Centor Criteria only includes 5 clinical characteristics of *S. pyogenes* infections, not including other important features, such as presence of palatal petechiae, sudden symptom onset, absence of oral ulcers or vesicles, presence of headache, absence of diarrhea, nausea and vomiting, among other findings. Thus, more important than just evaluating the final score of Modified Centor Criteria, it is necessary to consider the overall clinical picture so that we can improve the sensitivity and specificity of the clinical diagnosis of pharyngotonsillitis caused by *S. pyogenes*.

Other tests such as whole blood count and C-reactive protein (CRP) measurement are not specific to differentiate an infection caused by *S. pyogenes* from other infections. However, viral pictures usually occur with lymphocytosis and low levels of CRP, whereas bacterial pictures may show neutrophilia and higher CRP levels. Similarly, measurement of serum anti-streptolysin O (ASLO) levels is not useful for the diagnosis of acute infection, since detection in serum occurs only after the first week of infection, peaks between the 4th and 6th weeks, and may remain elevated for months after the infection.

### Treatment

There is great variability regarding the disease management recommendations among the several international consensuses, especially regarding the treatment of bacterial forms.^35^ Decades ago, it was believed that antibiotic use would be beneficial for all bacterial forms, aiming to abbreviate the odynophagia and fever symptoms. However, even in cases of bacterial pharyngotonsillitis, most of them (90% of cases) show complete and spontaneous resolution within 7 days.

Recent meta-analysis studies have shown that the use of antibiotics in bacterial cases actually shortens the duration of pain and significantly reduces the risk of developing rheumatic fever in more than 2/3 cases (RR = 0.22; 95% CI = 0.02–2.08). Similarly, it reduces the chance of developing AOM (RR = 0.30, 95% CI = 0.15–0.58), ABRS (RR = 0.48, 95% CI = 0.08–2.76) and peritonsillar abscess (RR = 0.15, 95% CI = 0.05–0.47) when compared with placebo. However, the pain improvement promoted by the antibiotic is very discrete, of around 16 h only, so the NNT is very high, not justifying its use as a generalized primary purpose in pain control or in the prevention of suppurative complications.^36^, ^37^ Thus, despite certain divergences between different international recommendations, there is evidence recommending the systematic treatment of all cases of pharyngotonsillitis caused by *S. pyogenes*, due to its good cost-benefit ratio for the primary prevention of rheumatic fever.

Thus, not every case of bacterial pharyngotonsillitis should be treated with antibiotics, except the most severe cases or cases of *S. pyogenes* etiology. It is worth mentioning that the treatment related to rheumatic fever prevention does not need to be implemented early (safety of up to 9 days to the beginning of the treatment), which allows the doctor to follow the patient's symptom evolution without making rushed decisions, or until the results of exams requested to complete the diagnosis and introduce the antibiotic are obtained. Table 8 summarizes the main clinical conditions in which the use of antibiotics is indicated in bacterial pharyngotonsillitis.

**Table 8:** Antibiotic use indications in bacterial pharyngotonsillitis.

Infections caused by *S. pyogenes* in regions where the risk of rheumatic fever is high
Presence of peritonsillar, parapharyngeal or retropharyngeal abscess
Very intense pain
Poor general status or toxemia
Presence of septic shock signs
Presence of dyspnea or stridor
Signs of dehydration
Severe comorbidities, such as decompensated diabetes and immunosuppression
Patients without improvement or worsening while using symptomatic treatment
Some pharyngotonsillitis caused by unusual agents, such as *C. diphtheriae*, *N. gonorrhoeae* or *Francisella tularensis*

**Figure 6:**
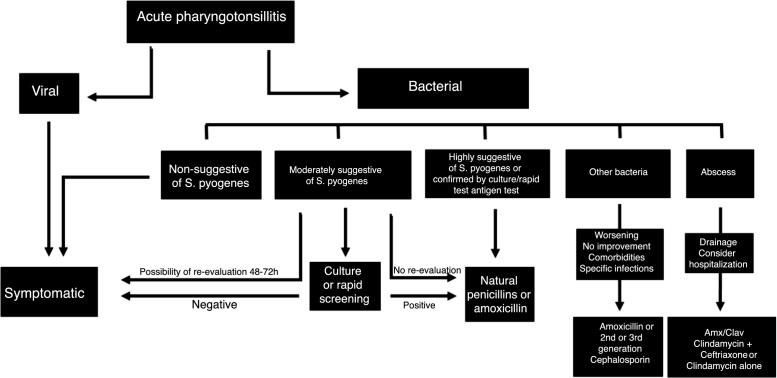
Treatment flowchart of patients with acute pharyngotonsillitis.

Regarding the use of antibiotics directed to *S. pyogenes* infections, phenoxymethylpenicillin (Penicillin V) or benzathine penicillin are considered the drugs of choice.^38^ Another good therapeutic option, considered by some guidelines as the first-choice drug, is amoxicillin at the dose of 50 mg/kg/day divided into 3 oral doses, for 10 days. It should be remembered that treatment with this drug for 7 days may not be effective in rheumatic fever primary prevention, because it does not eradicate *S. pyogenes* from the oropharynx. In case of patients allergic to penicillins, clarithromycin or erythromycin can be used.^39^, ^40^ In case of therapeutic failure with natural and/or synthetic penicillins, first-generation cephalosporins or clindamycin can be used. In some cases of bacterial pharyngotonsillitis with a presentation that is not characteristic of *S. pyogenes*, the possibility of other bacteria, such as Group C and G Streptococcus, *H. influenzae*, *Moraxella catarrhalis*, *S. aureus*, *Neisseria gonorrhoeae*, *Fusobacterium nucleatum* + *Borrelia vincentii* (Plaut-Vincent angina) must be considered. In those situations, when antibiotics are required, those with a broad coverage spectrum should be used, such as amoxicillin, amoxicillin-clavulanate or third-generation cephalosporins (Table 9). Fig. 6 summarizes the algorithm for the treatment of acute pharyngotonsillitis.

**Table 9:** Main antibiotics used in bacterial pharyngotonsillitis.

Antibiotic agent	Dose and posology	Time of treatment	Observations
*Indications for suspected or confirmed infection by S. pyogenes*			

Penicillin Benzathine	<27 kg: 600,000 IU, IM, single dose >27 kg: 1,200,000 IU, IM, single dose	Single dose	Drug of choice
Phenoxymethylpenicillin (Penicillin V)	<12 years: 90,000 IU/ kg weight/day, orally, 8/8 h >12 years: 200,000–500,000 IU, orally, 8/8 h	10 days	Drug of choice
Amoxicillin	≤30 kg: 50 mg/kg weight/day, orally, 8/8 h >30 kg: 500 mg, orally, 8/8 h or 875 mg, orally, 12/12 h	10 days	Drug of choice
Clarithromycin	Children: 15 mg/kg weight/day (maximum 250 mg/dose), orally, 12/12 h Adults: 250 mg, orally, 12/12 h or 500 mg (prolonged release drug), orally, 1×/day.	10 days	Indicated in the presence of allergy to penicillins
Erythromycin	Children: 30–50 mg/kg weight/day (up to 500 mg/dose), orally, 6/6 h Adults: 500 mg, orally, 6/6 h	10 days	Indicated in the presence of allergy to penicillins
Cefadroxil	Children: 25–50 mg/kg weight/day, orally, 12/12 h Adults: 500 mg, orally, 12/12 h	10 days	Indicated in therapeutic failure with penicillins
Cefalexin	Children: 25–50 mg/kg weight/day (up to 500 mg/dose), orally, 6/6 h Adults: 500 mg, orally, 6/6 h	10 days	Indicated in therapeutic failure with penicillins
Clindamycin	Children: 20–40 mg/kg weight/day, orally, 8/8 h, up to 300 mg/dose Adults: 300–600 mg, orally, 8/8 h	10 days	Indicated in therapeutic failure with penicillins


*Indications for other infectious agents*			

Amoxicillin	≤30 kg: 50 mg/kg weight/day, orally, 8/8 h >30 kg: 500 mg, orally, 8/8 h or 875 mg, orally, 12/12 h	10 days	
Cefuroxime	Children: 20 mg/kg weight/day, up to 250 mg/dose, orally, 12/12 h Adults: 500 mg/dose, orally, 12/12 h	10 days	
Amoxicillin-clavulanate	≤30 kg: 50 mg/kg weight/day (dose related to amoxicillin), orally, 8/8 h >30 kg: 500/125 mg, orally, 8/8 h or 875/125 mg, orally, 12/12 h	10 days	
Ceftriaxone	Children: 50–80 mg/kg weight/day, IV or IM, 1× a day Adults: 1–2 g/day, IV or IM, 1× a day	7 days	


*Indications in suppurative complications*			

Amoxicillin-clavulanate	≤30 kg: 50 mg/kg weight/day (equivalent dose of amoxicillin), orally or IV, 8/8 h >30 kg: 500/125 mg, orally, 8/8 h; 875/125 mg, orally, 12/12 h; 500/100 mg to 1000/200 mg, IV, 8/8 h	10–14 days	
Clindamycin	Children: 20–40 mg/kg weight/day, orally or IV, 8/8 h Adults: 300–600 mg/dose, orally or IV, 8/8 h	10–14 days	
Clindamycin + Ceftriaxone	Clindamycin, IV or orally, 8/8 h: Children: 20–40 mg/kg weight/day or Adults: 300–600 mg/dose + Ceftriaxone, IV or IM, 1× a day. Children: 50–80 mg/kg weight/day or Adults 1–2 g/day	10–14 days

## Discussion

The abusive and indiscriminate use of antibiotics on a global scale has led to a growing concern in all health area sectors. We are on the verge of experiencing a new era where banal infections can no longer be treated with antibiotics.^41^

Aiming to reverse this process, the WHO has started an intensive information campaign to reduce the risk of antibiotic resistance, and several countries have used guidelines, campaigns and even public health policies to reduce inappropriate antibiotic prescription, including Brazil, where antibiotics have only been sold under medical prescription since 2011. These measures were responsible for the immediate reduction in the sale of antibiotics in the country, but as early as March 2012, the records indicated that antibiotic sales were similar to those prior to the Regulations (around 8.7 million cases only in that month).^42^, ^43^ Other measures have been carried out by the Federal Government, such as information provided to the population by ANVISA. However, these measures are modest and have shown limited impact at national level.

In Brazil, there are also no studies that recorded the annual use of antibiotics, much less the diagnosis that led the physician to prescribe the drugs. Identifying the reasons why physicians prescribe antibiotics inappropriately is the first step toward an adequate intervention.^4^, ^44^, ^45^, ^46^, ^47^, ^48^

First, the indiscriminate use of antibiotics is related to the health professionals themselves and, second, to the patients.^45^ It is a concern that most of these protagonists do not show having knowledge about this important threat and dramatic reality of the abusive use of antibiotics.

Among the main reasons related to the prescribing physician are: years of practice, technical inexperience of the management of infectious diseases, complacency with guidelines and regulations and, especially, fear – either of losing the patient or having the patient develop any complications (which are known not be prevented by the use of antibiotics).

Even in developed countries, such as the US and Canada, it is estimated that approximately 30–50% of prescribed antibiotics are not in accordance with international recommendations. In Brazil and Latin America as a whole, there are no data on inadequate antibiotic use. However, it is believed that the reality of Latin America is similar to that of Eastern Europe, where the amount of prescribed antibiotics is even more alarming.^49^ As such findings are not restricted to developing or underdeveloped countries, it is believed that behavioral aspects are even more determinant than the cultural level of the society.^50^ Overall, there is a consensus that it is a complex context where aspects related to the history of professional training and prescription habits are very important and difficult to change.^51^ These conclusions are consistent with a society where the culture of fear, the practice of defensive medicine against lawsuits and even the expectation of faster cure using some type of medication, outweigh the scientific knowledge.

In one of the most successful international examples of a campaign for the rational use of antibiotics, Sweden has been able to reduce the number of antibiotic prescriptions associated with an outpatient visit to 318 per 1000 inhabitants by 2016.^52^ This number represents about a difference of almost 40% less in antibiotic prescriptions when compared to US numbers. Still in Sweden, the number of antibiotic prescriptions for acute otitis media decreased by 50% from 2000 to 2005, and even maintained gradual reductions to the present day. It should be noted that there are no records in this country of an increase in suppurative complications, such as mastoiditis,^53^ demonstrating that one of the conditions to attain success in this campaign was to convince physicians and patients that the non-prescription of antibiotics for most cases of AOM is not related to the increase in suppurative complications. This reduction in prescriptions was achieved in Sweden through interventions on prescriptions deemed inappropriate, either through educational measures for physicians and patients, continuing education for physicians, support for rapid diagnosis and clinical decision-making, and by convincing doctors to prescribe antibiotics “in case of symptom worsening”, the so-called “delayed prescription”.^4^, ^54^

Finally, educating the general population has been considered a key factor, so they can request antibiotics only when they are really feel the need for them. In this sense, the practice of delayed prescription, in which the doctor prescribes the medication “in case the patient really shows symptom worsening”, has reduced the number of antibiotic purchases.^47^, ^48^ However, for that to occur, the patients should be well advised on the negative consequences of antibiotic use, either for the patient or for the general population. For instance, by telling patients that the risk of negative consequences due to antibiotic use is much greater than the chance of complications due to upper airway bacterial infections. Alone, without contextualization and education, this measure has little positive repercussion.

We urgently need to reduce the alarming levels of unnecessary antibiotic prescriptions for upper airway infections (50%) as this practice has negative impacts on the patients (increasing the chances of side effects), on the health system (increasing the costs of our prescriptions) and on the general population (considerably increasing antibiotic resistance).

More than ever, it is necessary to reverse this situation, and for that purpose we need that ALL (doctors, patients, pharmaceutical industry, government, health systems, etc.) modify their actions and behaviors, with the common objective of providing a more precise and more conscious medical care to our population.

## Conflicts of interest

Eduardo Macoto Kosugi: speaker (Sanofi, Libbs); Fabrizio Ricci Romano: Advisory Board (Farmoquímica and Mylan); speaker (Takeda, Abbott e Glenmark); Edson I. Mitre: speaker (Sanofi); Edwin Tamashiro: speaker (Abbott). The other authors declare no conflicts of interest.
